# Learning to perform contrast-invariant cancellation of redundant stimuli

**DOI:** 10.1186/1471-2202-14-S1-P249

**Published:** 2013-07-08

**Authors:** Jorge F Mejias, Gary Marsat, Kieran Bol, Leonard Maler, Andre Longin

**Affiliations:** 1 Department of Physics, University of Ottawa, Ottawa, ON K1N6N5, Canada; 2 Department of Cellular and Molecular Medicine, University of Ottawa, Ottawa, ON K1H8M5, Canada; 3 Department of Biology & Center for Neuroscience, West Virginia University, Morgantown, WV 26506, USA; 4 Centre for Neural Dynamics, University of Ottawa, Ottawa, ON K1N6N5, Canada

## 

The cancellation of redundant information is a fundamental feature in many sensory systems. It constitutes, for instance, a key ingredient for the so called "cocktail party problem", in which a relevant signal has to be discerned and separated from other uninteresting stimuli in the auditory system [1]. However, the concrete mechanisms that the brain may employ to discriminate and cancel redundant information are presently unknown, and therefore candidate mechanisms have to be carefully studied.

Of special interest would be the cancellation mechanisms that are able to perform well under different realistic conditions, such as canceling redundant signals of different intensities. Properly addressing such a contrast-invariant cancellation mechanism could be beneficial to understand sensory processing in the visual system as well, since it is well known that contrast invariance is a prominent feature in neural circuits of primary visual areas [2].

In this work, we combine *in vivo *recordings and computational modeling to study a cerebellar-like circuit in the weakly electric fish which is known to perform cancellation of redundant stimuli. The cancellation is observed in a sub-population of neurons called superficial pyramidal (SP) neurons, which receive input both from electroreceptors encoding sensory input (constituting the feedforward pathway) and from a large population of granule cells (via parallel fibers) which are ultimately driven by electroreceptors as well (constituting the feedback pathway) [3,4]. Employing *in vivo *extracellular recordings, we observe contrast invariance in the cancellation of redundant stimuli in such a system. Our computational model, which improves previous modeling efforts [5,6], incorporates an heterogeneously-delayed feedback pathway, bursting dynamics in the SP neurons, burst-induced STDP observed in parallel fibers, precise input/output characteristics of the electroreceptors, and saturation properties of granule cells and parallel fibers. The predictions of this model are in agreement with our *in vivo *observations and provide an explanation for such a contrast-invariant cancellation.

In addition, the model: (I) gives insight on the activity of granule cells and parallel fibers involved in the feedback pathway, whose activity has not been recorded *in vivo *to date, and (II) provides a strong prediction on the time scale in which potentiation mechanisms in parallel fibers may occur. Our model also predicts that, in order to properly cancel redundant signals at the experimentally observed levels, the average contrast level that must drive the learning lies around contrast levels of 15%. Interestingly, contrast levels around this one are commonly found within the natural environment for communication signals in the weakly electric fish.
